# MitoMAMMAL: a genome scale model of mammalian mitochondria predicts cardiac and BAT metabolism

**DOI:** 10.1093/bioadv/vbae172

**Published:** 2024-11-05

**Authors:** Stephen Chapman, Theo Brunet, Arnaud Mourier, Bianca H Habermann

**Affiliations:** Aix-Marseille University, CNRS, IBDM UMR7288, Turing Center for Living Systems (CENTURI), Marseille 13009, France; Department of Biochemistry, Cell and Systems Biology, Institute of Systems, Molecular and Integrative Biology, The University of Liverpool, Liverpool L69 3BX, United Kingdom; Aix-Marseille University, CNRS, IBDM UMR7288, Turing Center for Living Systems (CENTURI), Marseille 13009, France; Université de Bordeaux, IBGC UMR 5095, Bordeaux 33077, France; Aix-Marseille University, CNRS, IBDM UMR7288, Turing Center for Living Systems (CENTURI), Marseille 13009, France

## Abstract

**Motivation:**

Mitochondria are essential for cellular metabolism and are inherently flexible to allow correct function in a wide range of tissues. Consequently, dysregulated mitochondrial metabolism affects different tissues in different ways leading to challenges in understanding the pathology of mitochondrial diseases. System-level metabolic modelling is useful in studying tissue-specific mitochondrial metabolism, yet despite the mouse being a common model organism in research, no mouse specific mitochondrial metabolic model is currently available.

**Results:**

Building upon the similarity between human and mouse mitochondrial metabolism, we present mitoMammal, a genome-scale metabolic model that contains human and mouse specific gene-product reaction rules. MitoMammal is able to model mouse and human mitochondrial metabolism. To demonstrate this, using an adapted E-Flux algorithm, we integrated proteomic data from mitochondria of isolated mouse cardiomyocytes and mouse brown adipocyte tissue, as well as transcriptomic data from in vitro differentiated human brown adipocytes and modelled the context specific metabolism using flux balance analysis. In all three simulations, mitoMammal made mostly accurate, and some novel predictions relating to energy metabolism in the context of cardiomyocytes and brown adipocytes. This demonstrates its usefulness in research in cardiac disease and diabetes in both mouse and human contexts.

**Availability and implementation:**

The MitoMammal Jupyter Notebook is available at: https://gitlab.com/habermann_lab/mitomammal.

## 1 Introduction

Mitochondria are essential organelles found in almost all eukaryotic cells and are indispensable for cellular bioenergetics, metabolism and homeostasis. One of their main objectives is to produce ATP through oxidative phosphorylation (OXPHOS). OXPHOS occurs within the inner mitochondrial membrane, where electrons are shuttled along an Electron Transport Chain (ETC) mediated by the mobile electron carriers, Coenzyme Q (CoQ) and cytochrome C. Electron transfer through each complex is coupled to proton translocation from the mitochondrial matrix to the intermembrane space which generates a Proton Motive Force (PMF) across the inner membrane that is used by ATP-synthase, to phosphorylate ADP to ATP (Hatefi 1985). Several other metabolites are also directly oxidized by the ETC by reducing CoQ. In mammals, these include the mitochondrial glycerol-3-phosphate dehydrogenase (G3PDH) (Mráček *et al.* 2013), dihydroorotate dehydrogenase (DHODH) (Molinié *et al.* 2022), proline (Tanner *et al.* 2018, Pallag *et al.* 2022), and the electron transfer flavoprotein dehydrogenase (Lenaz *et al.* 2007), the first step of mitochondrial fatty acid oxidation.

Apart from the production of cellular energy, mitochondria are integral to various cellular and metabolic processes including pacing organism-specific development rates (Diaz-Cuadros *et al.* 2023), apoptosis (Suen *et al.* 2008), calcium signalling (Nicholls 2005), and regulating reactive oxygen species (ROS) production, which itself is an important secondary messenger (Forman *et al.* 2010). Mitochondria also generate metabolic intermediates crucial for biosynthetic pathways and redox regulation (Borst 2020). Due to their central roles in cellular metabolism, signalling and bioenergetics, dysregulated mitochondrial metabolism is associated with various human diseases, emphasizing their critical role in maintaining cellular health (Liesa *et al.* 2009). Understanding the intricacies of mitochondrial metabolism is therefore essential for advancing knowledge of cell biology, physiology, and medicine.

### 1.1 Tissue-specificity of mitochondrial structure and content

Mitochondrial structure (Kuznetsov *et al.* 2009, Liesa *et al.* 2009), and proteome content vary across tissues (Calvo and Mootha 2010, Williams *et al.* 2018, Hansen *et al.* 2024). Considering the metabolic roles played by proteins, proteomic changes would reroute metabolism to sustain different biological objectives in various cellular contexts. Therefore, mitochondrial metabolism and function are highly specialized to meet diverse cellular functions and bioenergetic needs. This is strongly evidenced in cardiomyocytes which are responsible for the control of the rhythmic beating of the heart and rely heavily on ATP to achieve maximal cardiac output (Karbassi *et al.* 2020). Brown Adipose Tissue (BAT) is a specialized type of adipose tissue with unique mitochondrial properties that permit thermogenic heat generation (Flatmark and Pedersen 1975, Takeda *et al.* 2023). One key characteristic of brown adipocyte mitochondria is a high abundance of uncoupling protein 1 (UCP1), which is responsible for uncoupling OXPHOS from ATP production (Wang *et al.* 2019, Hansen *et al.* 2024). This uncoupling leads to the dissipation of the PMF across the inner mitochondrial membrane as heat, a process crucial for non-shivering thermogenesis (Nicholls 1979, 2021), thus highlighting an alternate biological objective of the mitochondria within BAT. A better understanding of mitochondrial metabolism could, for instance, help reduce the prevalence of metabolic diseases in cardiac and other chronic metabolic diseases like diabetes. To give just one example, dysregulated ATP synthase activity following activation of inhibitory factor 1 (IF1) is implicated with a wide range of metabolic diseases including diabetes (Sergi *et al.* 2019, Wei *et al.* 2024).

### 1.2 System-level modelling to gain in-depth insight into tissue-specific mitochondrial metabolism

Systems-level modelling of mitochondrial metabolism is essential to provide novel and testable model-driven insights into mitochondrial function and disease (Ben Guebila and Thiele 2021, Heinken *et al.* 2021, Wagner *et al.* 2021, Tomi-Andrino *et al.* 2022). Flux Balance Analysis (FBA) is a computational method that implements linear programming in conjunction with a metabolic reconstruction to predict metabolic fluxes on the systems level (Orth *et al.* 2010, Sahu *et al.* 2021, Westerhoff 2023). By integrating existing knowledge of mitochondrial biology into such a modelling framework, researchers can specifically analyse mitochondrial metabolism. Omics data, such as transcriptomics or proteomics can be integrated into a metabolic reconstruction using a variety of methods such as E-Flux (Colijn *et al.* 2009)/E-Flux2 (Kim and Lun 2014, Kim *et al.* 2016), to produce context-specific metabolic models. Thus, metabolic modelling can facilitate a better understanding of the metabolic differences between tissues or disease conditions.

The mitochondrial metabolism of humans and mice is included in several metabolic reconstructions. Recon 1 was the first generic human metabolic model (Duarte *et al.* 2007). Recon 1 has been updated to Recon R2, which included additional biological information and the correction of various modelling errors such as Recon 1’s inability to correctly predict realistic ATP yields (Thiele *et al.* 2013). Recon R2 was subsequently upgraded to Recon 3D, which includes a total of 13 543 metabolic reactions and extensive human gene-product-reaction (GPR) associations (Brunk *et al.* 2018). In parallel to the recon lineage of human metabolic models, a Human Metabolic Reaction series (HMR1 and 2) were developed and used to specifically model a human adipocyte and a hepatocyte, respectively, containing 6160 and 7930 metabolic reactions. Metabolic information from HMR2 was then complemented with information from Recon 3D to produce a unified metabolic model of human metabolism, called Human1, now containing over 13 000 reactions, 10 000 metabolites and 3625 genes. Human1 has since been used as a template to produce specific genome scale metabolic models of the fruit fly, worm, zebrafish, rat, and mouse using orthologue mapping and identification of species-specific metabolism using literature and databases. The mouse specific metabolic model remains the most concise mouse metabolic model to date and contains more metabolic reactions than its predecessor, iMM1865, which was produced using a top-down orthology-based methodology by mapping human genes of Recon 3D to mouse genes (Khodaee *et al.* 2020).

One challenge facing predictive modelling at the genome-scale level is that large models are more error prone than smaller models. This is a consequence of missing knowledge and/or incorrect annotation. For example, the reconstruction and interpretation of the GPR rules adds uncertainty to the annotation process, and the process of constructing genome scale models involves gap filling that connects dead-end metabolites using reactions inferred from other models. This is essential to satisfy steady-state metabolism, however, this step is inherently uncertain as the new reactions might not be supported by the genome (Bernstein *et al.* 2021). Other sources of error include missing information relating to metabolite mass due to incorrect formulas (Chapman *et al.* 2017), incorrect parameterization of reaction directionality constraints and issues relating to the incorrect compartmentalization of reactions and metabolites. These uncertainties accumulate and can account for mispredictions that include the incorrect operation of metabolite shuttles and the reversal of proton pumping (Smith *et al.* 2017), and the generation of infeasible metabolic cycles. As such, using large genome-scale models to specifically predict mitochondrial metabolism can, therefore, result in mispredictions (Fritzemeier *et al.* 2017, Ong *et al.* 2020, Bi *et al.* 2022, Karp *et al.* 2023).

Several concise models of human mitochondrial metabolism exist (Smith and Robinson 2011, Smith *et al.* 2017), with MitoCore representing the latest and most comprehensive model of human cardiomyocyte mitochondrial metabolism (Smith *et al.* 2017). MitoCore includes the ETC within its reconstruction and can accurately model the PMF associated with ATP production. This model has successfully been applied to model fumarase deficiency (Smith *et al.* 2017), impaired citrate import (Majd *et al.* 2018) and predicted accurate respiratory quotients on glucose and palmitate substrates (Cabbia *et al.* 2021) which demonstrates MitoCore’s potential to model human cardiomyocyte mitochondrial metabolism.

Mice are often employed as a model organism in mitochondrial research due to their highly similar structure, function and genetic homology with human mitochondria. This similarity makes mice a valuable model system for advancing our understanding of mitochondrial biology, mitochondrial dysfunction and disease, and for exploring potential interventions for mitochondrial-related disorders in humans. Because of these similarities, mouse mitochondrial metabolism is routinely compared to human mitochondrial metabolism in diverse biological contexts (Porter *et al.* 2016, Diaz-Cuadros *et al.* 2023). Despite the prevalence of mice *in vivo*, *in vitro*, and *in silico* models, there are no concise *in silico* models of mouse mitochondrial metabolism.

To address this limitation, and to valorize the opportunity presented by mitochondrial similarity, in this work, we have created mitoMammal, a mitochondrial metabolic network which can be used for constraint-based metabolic modelling of human and mouse mitochondria. Importantly, mitoMammal can be contextualized with omics data emerging from humans or mice, allowing for the capacity to model the metabolism of both species. To demonstrate this novelty, we have integrated mitochondrial transcriptomic data from Brown Adipocytes (BAs), and then mitochondrial proteomic data from mice BAT and cardiac tissues. We found that integrating proteomic and transcriptomic data from humans and mice into mitoMammal predicted proline dehydrogenase and G3PDH reduction of CoQ, the export of hexadecanoic acid from BAT tissue, and glycine import to sustain cardiomyocyte metabolism.

## 2 Methods

### 2.1 Conversion of mitoCore into mitoMammal

**Figure 1.**
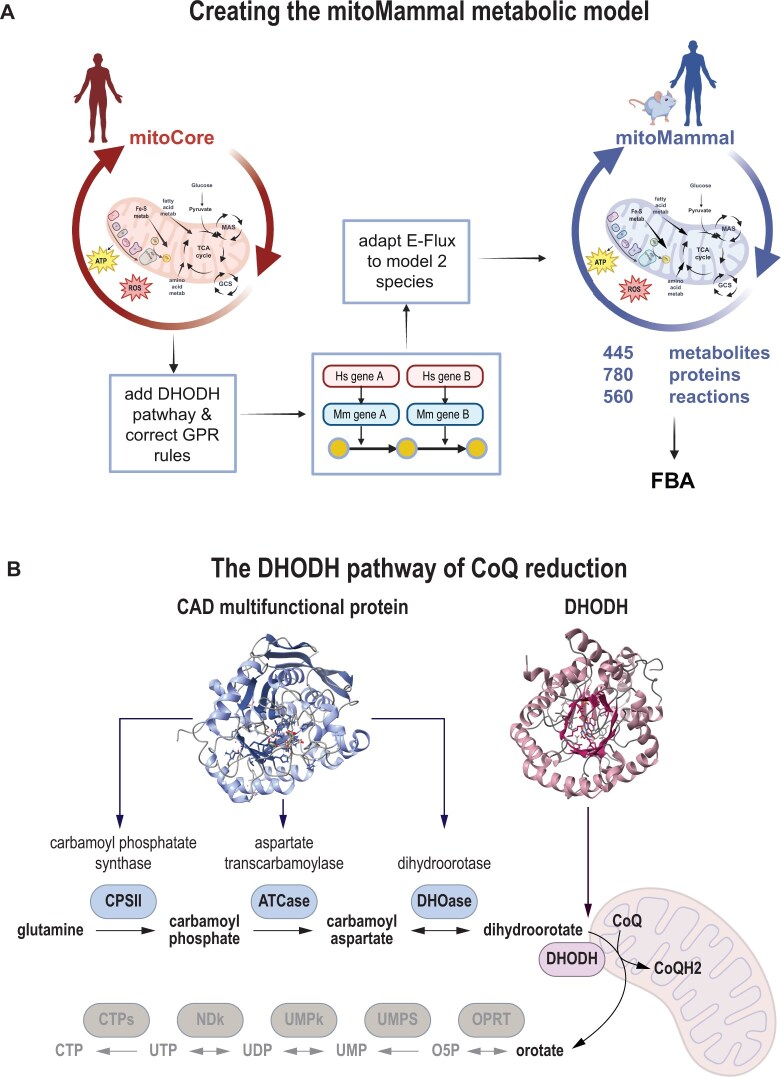
Conversion of MitoCore to mitoMammal. (A) Workflow for the construction of the mitoMammal metabolic network. (B) The DHODH pathway of CoQ reduction was added to the mitoMammal mitochondrial metabolic model. Figure part (A) created with BioRender.com.

To build the mitoMammal mitochondrial metabolic model, we identified the mouse orthologues of MitoCore’s GPRs using BioMart (Kasprzyk 2011) and the ENSEMBL database (Martin *et al.* 2023), as well as orthology information stored within mitoXplorer2 (Yim *et al.* 2020, Marchiano *et al.* 2022). This resulted in 389 mouse orthologues out of the original complement of 391 MitoCore genes (Supplementary Table S1). The set of mitoCore GPR rules was compiled to their corresponding logical expressions for mitoMammal based on orthology relations between human and mouse genes. A summary of mitoMammal construction is represented in Fig. 1A (see also Supplementary Table S1). Gene modifications in the mouse version of the metabolic model reconstruction are listed in Table 1 and discussed below.

**Table 1.** Changes applied from human to mouse in mitoMammal.

Human gene	Action in mitoMammal
G6PD	renamed as G6pd2
ATP5F1	renamed as Atp5pb
ATP5I	renamed as Atp5k
Pc	renamed as Pcx
NME2	renamed as Gm20390
FH	renamed as Fh1
ACSM6	deleted—no evidence in mouse
OGDH	renamed as Dhtkd1
GLUD2	deleted—no evidence in mouse
DHFRL1	renamed as Dhfr
GCAT	has two mouse orthologues; so ENSMUSG00000116378 was complemented with ‘OR’ ENSMUSG00000006378
SLC25A6	deleted—not found in mouse

### 2.2 DHODH expansion

The discovery that dihydroorotate can reduce CoQ in mouse mitochondria suggests that this is a conserved feature of all mammalian mitochondria (Molinié *et al.* 2022). MitoCore (Smith *et al.* 2017) was missing the reduction of CoQ by DHODH within the *de novo* pyrimidine synthesis pathway, while it contained glutamine metabolism, which is the starting substrate for this pathway. Initially, glutamine is converted to carbamoyl phosphate facilitated by carbamoyl phosphate synthase. Carbamoyl phosphate is then metabolized to carbamoyl aspartate through the activity of aspartate carbamoyltransferase, which is subsequently metabolized into dihydroorotate by the enzyme dihydroorotase (Fig. 1B). In mammals, these three enzymes are part of a single multifunctional protein abbreviated as CAD (Carbamoyl Aspartate Dihydroorotase). Dihydroorotate then reduces CoQ to produce orotate, facilitated by the enzyme dihydroorotate acid dehydrogenase (DHODH) that sits at the surface of the outer mitochondrial membrane. As such, orotate is never imported into the mitochondria and remains cytoplasmic (Zhou *et al.* 2021). We included these metabolic reactions and new metabolites in mitoMammal. Orotate removal from the model was implemented by the addition of a demand reaction to maintain flux consistency. In total, five new reactions were added that incorporate four new metabolites and two new genes.

### 2.3 Correction of the mitoMammal model based on gene expression data

Because the ETC is at the heart of mitoMammal, we closely inspected the GPR rules of the 5 ETC complexes and found a number of paralogous genes that were bound by an AND relationship. Furthermore, by integrating gene expression data, we observed that fluxes of Complex I and IV of the respiratory chain in the mitoMammal model, and hence also in MitoCore, were strongly reduced, or even shut down completely. We analysed the gene expression patterns of the paralogs and then corrected paralogous gene pairs to an OR relationship. These specifically included (mentioned as human paralog and mouse paralog pairs): Complex I: Ndufb11b/Ndufb11b [ENSMUSG00000031059/ENSMUSG00000061633 (mouse only)]. NDUFA4/NDUFA4L2 (ENSG00000189043/ENSG00000185633) and Ndufa4/Ndufa4l2 (ENSMUSG00000029632/ENSMUSG00000040280). Complex IV: COX4I1/COX4I2 (ENSG00000131143/ENSG00000131055) and Cox4i1/Cox4i2 (ENSMUSG00000031818/ENSMUSG00000009876). COX6A1/COX6A2 (ENSG00000111775/ENSG00000156885) and Cox6a1/Cox6a2 (ENSMUSG00000041697/ENSMUSG00000030785). COX6B1/COX6B2 (ENSG00000126267/ENSG00000160471) and Cox6b1/Cox6b2 (ENSMUSG00000036751/ENSMUSG00000051811). COX7A1/COX7A2 (ENSG00000161281/ENSG00000112695) and Cox7a1/Cox7a2/Cox7a2l (ENSMUSG00000074218/ENSMUSG00000032330/ENSMUSG00000024248). COX8A/COX8C (ENSG00000176340/ENSG00000187581) and CoX8a/Cox8c (ENSMUSG00000035885/ENSMUSG00000043319). We furthermore added UCP1 [ENSG00000109424, Ucp1 in mouse (ENSMUSG00000031710)] to the model, as this gene was not included in the original MitoCore model due to the model’s specificity for heart metabolism.

### 2.4 Update to SBML3 (version 1)

The original MitoCore model was encoded using Systems Biology Markup Language (SBML) level 2 annotation. We updated the mitoMammal to the most recent, relevant specification of SBML level 3 (version 1) (Keating *et al.* 2020) and validated the model for correctness using the online SBML validation tool [https://synonym.caltech.edu/validator_servlet/index.jsp; (Lister *et al.* 2007)].

### 2.5 Adapting the E-Flux algorithm for mitoMammal

Because the MitoMammal metabolic model can integrate -omics data from two, instead of one species, we modified the E-Flux algorithm by allowing the user to select the organism the data originates from. The adapted algorithm then uses -omics data to constrain the reactions specific to the chosen species. All other features of the original E-Flux method were maintained as in the original description of the algorithm (Colijn *et al.* 2009). The adapted E-Flux algorithm was used to constrain mitoMammal with mouse proteomic data and transcriptomic data from humans.

The adapted E-Flux algorithm first selects the data for the genes or proteins that are in the model and scales everything between 0 and 1 by dividing by the 90th percentile and values greater than 1 are capped at 1. These scaled values are then used to calculate the upper-bound of each reaction based on the GPR. For reactions that require multiple genes that all have to be expressed and are thus linked by an AND relationship, we assume as the upper bound the value of the gene with lowest expression. In case of an OR relationship between genes, each individual gene can contribute to the reaction and the sum of their values is used as the upper bound. This algorithm corresponds to the original E-Flux algorithm (Colijn *et al.* 2009) and has been adapted in python to work with COBRApy (see Supplementary Fig. S1 for a workflow). The adapted E-Flux algorithm was used to constrain mitoMammal with mouse proteomic data and transcriptomic data from human and mouse.

### 2.6 Mouse proteomic data

For mouse simulations, we integrated proteomic data from a recent study that extracted the mito-proteomes of isolated mitochondria from a range of mouse tissues (Hansen *et al.* 2024). Normalized protein counts of cardiac and brown adipose tissue were scaled between 0 and 1. For cardiac tissue, we optimized ATP hydrolysis and for BAT simulations, we optimized the UCP reaction considering its essential role in producing non-shivering heat in this tissue.

### 2.7 Human transcriptomic data

We used normalized RNA-sequencing data from Rao (Rao *et al.* 2023) (GEO dataset GSE185623) to model an *in vitro* differentiated hiPSC-derived brown adipocyte (BA). Normalized read counts were scaled between 0 and 1. The UCP reaction was chosen to be optimized considering the essential role that UCP1 plays in uncoupling ETC from ATP synthesis in BAs which is a prerequisite for producing non-shivering heat.

### 2.8 Flux balance analysis

Parsimonious FBA was performed using Python (version 3.8.5) in conjunction with the COBRApy toolbox (Ebrahim *et al.* 2013), using the default ‘GLPK’ solver.

The mitoMammal metabolic model, along with Jupyter notebooks and data used in this work are available at: https://gitlab.com/habermann_lab/mitomammal.

## 3 Results

### 3.1 The mitoMammal metabolic network for human and mouse mitochondrial metabolism

**Figure 2.**
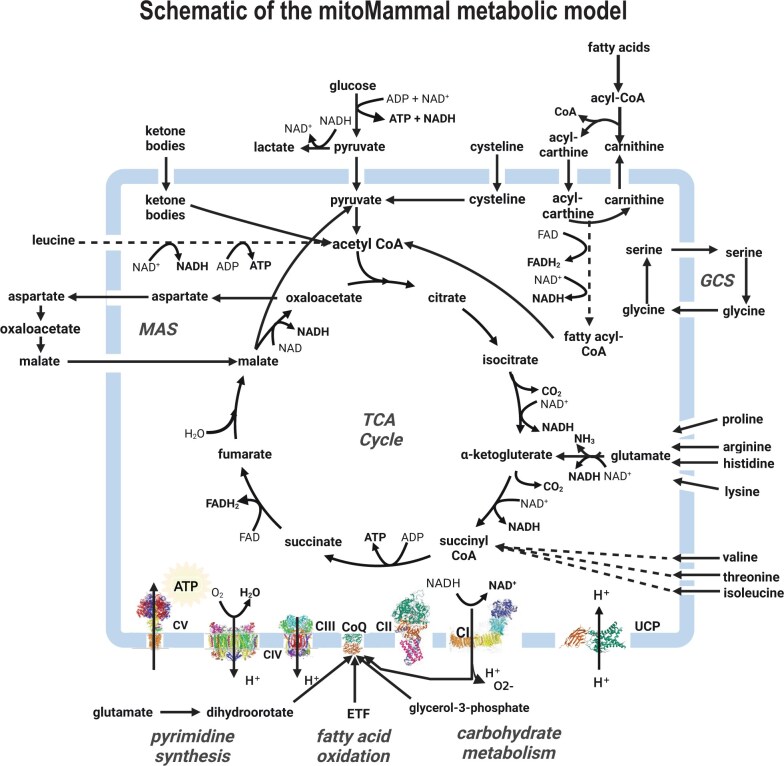
MitoMammal metabolic reconstruction consists of 780 genes (human and mouse orthologs) encoding 560 metabolic reactions. Initially constructed from MitoCore (Smith *et al.* 2017), it was expanded to include DHODH reduction of CoQ and then supplemented with mouse orthologous genes. MitoMammal can be used for detailed mitochondrial metabolic studies of both human and mouse. Figure created with BioRender.com.

This work aimed to produce a generic mammalian metabolic model of mitochondrial metabolism that incorporates new knowledge on CoQ fuelling. We first translated the genes from the human MitoCore model into mouse genes using orthology inference to create the basic mitoMammal model. Key metabolic pathways that include the TCA cycle, the Malate Aspartate Shuttle (MAS); OXPHOS and ATP synthesis; the Glycine Cleavage System, the proline cycle and fatty acid oxidation were also retained from the original model. MitoMammal now includes *de novo* pyrimidine synthesis from glutamate leading to the reduction of the CoQ complex by the enzyme DHODH. MitoMammal contains 780 genes encoding 560 metabolic reactions that involve 445 metabolites. The complete lists of reactions, metabolites, and associated fluxes from each simulation are available in Supplementary Table S1a–c, respectively. The core metabolism and bioenergetics with associated import/export reactions of the model are depicted in Fig. 2.

MitoMammal is based on MitoCore, a human specific cardiomyocyte mitochondrial model. MitoMammal was first tested on its ability to correctly produce accurate ATP levels from glucose oxidation. All nutrient input reactions except glucose and oxygen were constrained to zero to reflect aerobic glycolytic conditions. Maximization of ATP hydrolysis was used as the objective function for these simulations and the model was then optimized using parsimonious FBA for all simulations reported in this work. As expected, MitoMammal correctly predicted the production of 31 molecules of ATP from 1 molecule of glucose (Supplementary Fig. S2).

### 3.2 Modelling cardiac mitochondrial metabolism by integrating mouse proteomic data of cardiac tissue

**Figure 3.**
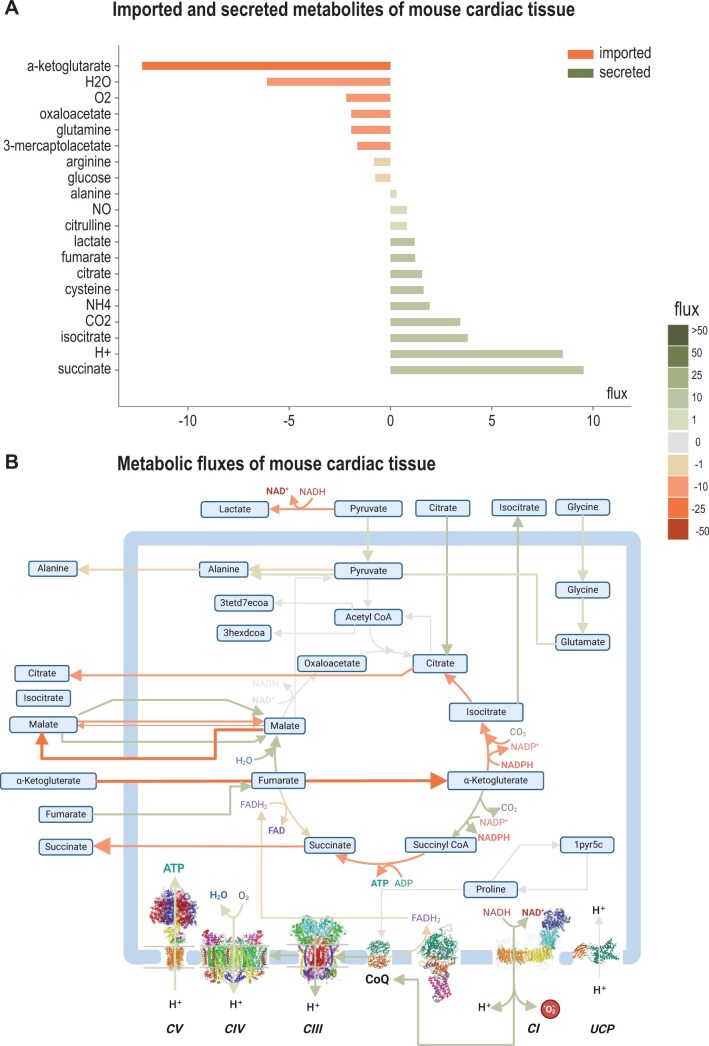
Metabolic flux prediction from mitoMammal following integration of mouse proteomic data isolated from cardiac tissue and optimising ATP production. (A) Imported and secreted metabolites were predicted to result in steady-state mitochondrial fluxes of mitochondria isolated from mouse cardiac cells following the integration of proteomic data. Imported metabolites by convention, are associated with negative fluxes while secreted metabolites are described with positive fluxes. (B) The predicted flux distribution describes an import of glycine into the mitochondria, along with the import of citrate, fumarate and ɑKG. *De novo* fatty acid synthesis occurs as a result of pyruvate conversion into acetyl-CoA. The colour code of the fluxes is given at the right-hand side of the figure. Figure part (B) created with BioRender.com.

To demonstrate mitoMammal’s ability of modelling mouse cardiac mitochondrial metabolism, we integrated proteomic data harvested from mitochondria isolated from mouse cardiac tissue and optimized ATP hydrolysis. This resulted in 330 constrained reactions out of the complement of 560 reactions. In satisfying the objective subject to these constraints, mitoMammal predicted the import of ɑKG, H_2_O, oxygen, oxaloacetate, glutamine, 3-mearcaptoacetate, and glucose. The model also predicted the export of alanine, NO, citrulline, lactate, fumarate, citrate, cysteine, NH_4_, CO_2_, isocitrate, hydrogen, and succinate (Fig. 3A).

Flux predictions (Fig. 3B) revealed that the flux of pyruvate emerging from glycolysis was partitioned between lactate production in the cytoplasm, and pyruvate import into the mitochondria. This is in agreement with the literature that reports a mitochondrial involvement of lactate production (Flick and Konieczny 2002). It is now understood that the shuttle of lactate from and between cardiomyocytes to other cells facilitates lactate supply to cells in need of lactate, and acquired lactate plays a plethora of important roles such as cell signalling (Daw *et al.* 2020), the regulation of cell proliferation (Liu *et al.* 2023) and development of organs and in the coordination of vascular development and progenitor cell behaviour in the developing mouse neocortex (Dong *et al.* 2022). TCA cycle fluxes were sustained by the import of citrate, ɑKG, fumarate, and malate. Glycine was imported into the mitochondria and converted to glutamate.

### 3.3 Modelling mouse BA metabolism by integrating mouse proteomic data with mitoMammal

**Figure 4.**
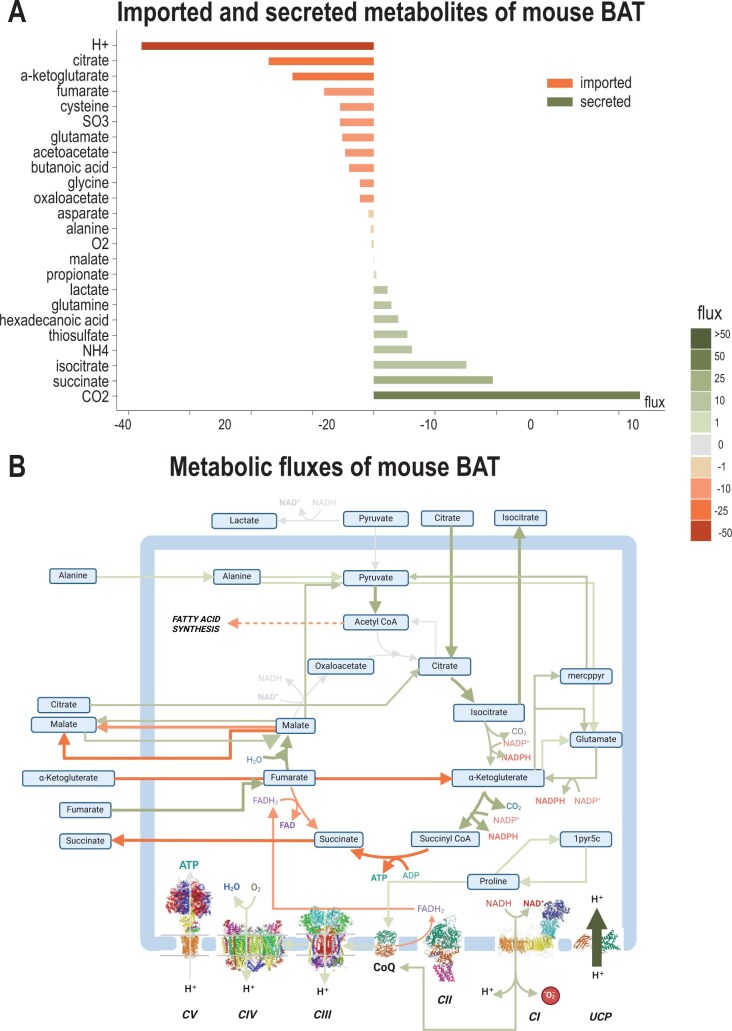
Proteomic data of mitochondria isolated from murine BAT was used to constrain mitoMammal. (A) Imported and secreted metabolites were predicted to result in steady-state mitochondrial fluxes of mitochondria isolated from mice BAT cells following the integration of proteomic data. Imported metabolites by convention, are associated with negative fluxes while secreted metabolites are described with positive fluxes. (B) As a result of optimizing the UCP reaction, the ETC is disengaged from ATP production emerging from OXPHOS. Steady-state TCA cycle fluxes are established by the import of alanine, citrate, fumarate, and ɑKG. *De novo* fatty acid synthesis occurs as a result of pyruvate conversion into acetyl-CoA. mitoMammal also predicts the reduction of CoQ by proline, which is produced by 1-Pyrroline-5-Carboxylate (1pyr5c). The colour code of fluxes is given on the right side of the figure. Figure part (B) created with BioRender.com.

We next wanted to show the predictive power and usability of mitoMammal to predict mouse mitochondrial metabolism in a BA cell by integrating mitochondrial proteomic data extracted from brown adipose tissue (BAT) (Hansen *et al.* 2024). Following data integration, we then optimized flux towards the UCP reaction. From the model’s complement of 560 reactions, our modified E-Flux algorithm constrained 329 reactions. In order of decreasing flux magnitude, mitoMammal predicted the import of hydrogen, citrate, ɑKG, fumarate, cysteine, sulfate, glutamate, acetoacetate, butanoic acid, glycine, oxaloacetate, aspartate, alanine, and O_2_. Secreted metabolites consisted of malate, propionate, lactate, glutamine, hexadecenoic acid, thiosulfate, NH4, isocitrate, succinate, and CO_2_ (Fig. 4A).

In this simulation, citrate, fumarate, ɑKG and to a lesser extent, malate were predicted to be imported into the mitochondria to establish steady-state TCA cycle fluxes. Imported ɑKG was metabolized into succinyl-CoA within the TCA, and into 3-Mercaptopyruvic acid (mercppyr) exterior of the TCA cycle which then fed into pyruvate metabolism. Pyruvate metabolism was also established by the import of alanine and with conversion of malate into pyruvate. The majority of pyruvate was converted into acetyl-CoA and with the addition of citrate, channelled flux towards fatty acid synthesis and the export of hexadecanoic acid. Citrate was imported into mitochondria and assimilated into the TCA cycle, and upon conversion to Isocitrate, was then partially exported from mitochondria.

Complex I (CI) was predicted to be reduced by NADH emerging from the TCA cycle, which injected electrons into the ETC and reduced the CoQ complex. MitoMammal also predicted the reduction of CoQ with proline via the proline dehydrogenase reaction (PROD2mB, encoded by the PRODH gene). CII was predicted to operate in reverse and reduce fumarate leading to succinate production and its subsequent export. From CoQ, electrons were passed along the ETC towards CIII and CIV which produced PMF, however, ATP synthase (CV) in this situation was predicted to be inactive, and the UCP reaction was active and carried the largest flux in this simulation. Mitochondrial uncoupling via UCP1 is a process that expends energy by oxidizing nutrients to produce heat, instead of ATP. To better understand the role played by the UCP reaction in BAT tissue, we next examined the reactions that would consume the newly uncoupled protons after their re-entry into the mitochondria to identify novel functionalities of the UCP reaction in BAT. Twenty proton-consuming reactions were identified and are shown in Supplementary Table S2.

The largest subset of these reactions performed metabolism of fatty acid and consisted of MECR14C and MECR16C which are responsible for fatty acid elongation of 3-Hydroxy Tetradecenoyl-7 Coenzyme A and 3-Hydroxyhexadecanoyl Coenzyme A respectively. Also belonging to this group were the reactions MTPC14, MTPC16, r0722, r0726, r0730, r0733, and r0791 and all performed fatty acid oxidation roles and released NADH within the mitochondria. The remaining reactions of this subset (r0633, r0638, r0735) all consumed mitochondrial NADP. 4 more reactions performed metabolite transport functions with a citrate-carrying reaction (r0917b) carrying the greatest flux of this analysis. This reaction exports isocitrate and protons in exchange for citrate import. The model predicted flux associated with the characterized mitochondrial carrier responsible for the export of phosphate and photons (Plt2mB) out of the mitochondria. The citrate-malate antiporter (CITtamB) was also predicted to be active in exporting malate and protons in exchange for the import of citrate. Uncoupled protons were predicted to leak out of the mitochondria, facilitated by the Hmt reaction.

A further subset of 3 reactions were implicated with amino acid metabolism. Within this subset, the reaction to carry the largest flux was 3-Mercaptopyruvate: Cyanide Sulfurtransferase (r0595m) in mouse BAT, which converts mercaptopyruvate and sulfate into pyruvate and thiosulfate. Also within this subset is the P5CRxm that involves the production of proline, and finally the methylmalonyl Coenzyme A decarboxylase reaction (MMCDm) which converted methylmalonyl-CoA into propionyl-CoA. The remaining reaction predicted to metabolize uncoupled protons was the CI reaction of the OXPHOS subsystem.

### 3.4 Modelling brown adipocyte metabolism in humans

**Figure 5.**
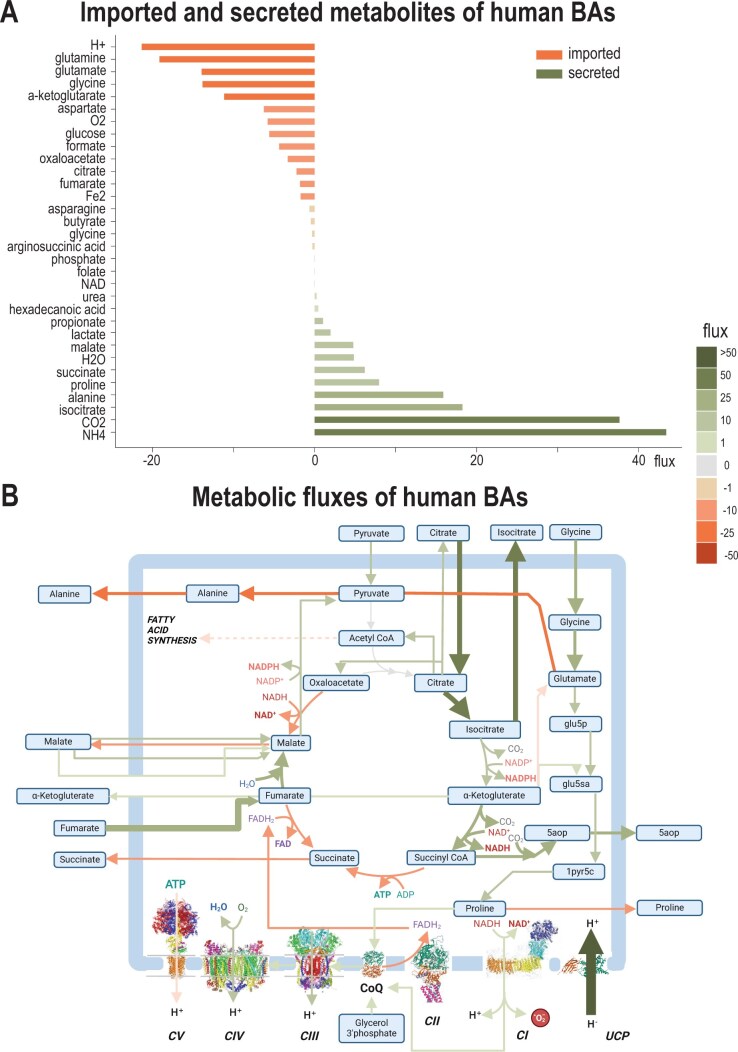
Flux distribution following the integration of transcriptomic data from a human brown adipocyte into mitoMammal. (A) Imported and secreted metabolites predicted from steady-state mitochondrial fluxes of mitochondria from a human brown adipocyte following the integration of transcriptomic data. Imported metabolites by convention, are associated with negative fluxes while secreted metabolites are described with positive fluxes. (B) Metabolic fluxes are predicted to activate the UCP reaction which has the effect of uncoupling the ETC from OXPHOS to support steady-state metabolism during thermogenesis. Import of glycine, fumarate and ɑKG were predicted to sustain flux through the TCA cycle. Acetyl-CoA emerging from citrate import was then predicted to feed into *de novo* fatty acid synthesis. Metabolites exported out of the mitochondria were 5-aminolevulinic acid (5aop), proline, succinate, malate and alanine. Fluxes are highlighted in arrow thickness and colour; green colours: positive fluxes; orange colours: negative fluxes. Figure part (B) created with BioRender.com.

Next, we wanted to demonstrate mitoMammal’s ability of modelling human mitochondrial metabolism. To this end, we integrated transcriptomic data (Rao *et al.* 2023) from a brown adipocyte (BA) that was differentiated from an IPSC and optimized the UCP reaction. This resulted in constraining 488 reactions out of the complement of 560 reactions. Analysis of the resulting fluxes revealed that 15 metabolites were predicted to be imported into mitoMammal to support steady-state mitochondrial BA metabolism. Similar to the mouse model, H+, glutamate, cysteine, aKG, aspartate, O_2_, oxaloacetate, fumarate, and glycine were imported, however, with different magnitudes. The largest flux was again associated with H+ import. In addition, glutamine, glucose, formate, citrate, Fe_2_, and argininosuccinate were imported. Similar excreted metabolites included NH_4_, CO_2_, isocitrate, malate, lactate, propionate, and hexadecanoic acid. Opposed to the mouse model, alanine was exported, and not imported. In addition, the human model secreted proline, H_2_O, urea, NAD, folate, and phosphate (Fig. 5A).

Similar to the mouse simulation (Fig. 4B), the import of citrate, fumarate, ɑKG and malate were predicted to contribute to sustaining steady-state TCA cycle fluxes. In human BAs, pyruvate emerging from glycolysis was predicted to be imported into the mitochondria and converted to alanine which was then, opposite to mouse BAs, exported out of the mitochondria. Citrate played a dual role and was also metabolized into acetyl-CoA which subsequently fed into endogenous fatty acid synthesis via acetyl-CoA, which agrees with the literature that describes mammalian BAT as possessing high endogenous fatty acid synthesis activity (Calderon-Dominguez *et al.* 2016, Schlein *et al.* 2021) (Fig. 5B). In particular, the model predicted the synthesis and export of hexadecanoic acid and 5-Aminolevulinate (5aopm) from the mitochondria.

Fluxes through ETC were similar to the mouse BAT simulation, except now, CV which in this simulation was predicted to operate in reverse and consumed ATP. Similar to before, CII was predicted to operate in reverse. The model furthermore predicted the reduction of CoQ with proline via the proline dehydrogenase reaction (PROD2mB, encoded by the PRODH gene). PRODH forms part of the proline cycle that regenerates proline via pyrroline-5-carboxylate which, in contrast to the mouse model, leads to the subsequent export of proline. In addition, we predicted the reduction of CoQ by G3PDH, which was not predicted in the mouse simulation. The reaction carrying the greatest flux in this simulation was again the UCP reaction which uncoupled the ETC from ATP production. We then analysed all proton consumption reactions predicted to be active as a consequence of optimal UCP1 activity. All reactions that carry a flux greater than 0.01 are also shown in Supplementary Table S2.

As with the previous simulation of mouse BAT, the reaction to carry the greatest flux was attributed to the citrate-carrying reaction (r0917b). The largest subset of reactions was also implicated with the same fatty acid metabolic reactions as reported in the previous simulation, however, carrying much reduced predicted fluxes. The next subset of reactions again, all involved transport functions with the first that exported phosphate and protons (Plt2mB) out of the mitochondria, and the citrate-malate antiporter (CITtamB). Uncoupled protons were also predicted to leak out of the mitochondria, as facilitated by the proton-transport reaction. The final reaction predicted to be active in this simulation, which also was predicted to be active in the previous simulation, was the Pyrroline-5-Carboxylate Reductase reaction (P5CRxm). Similarly to the mouse BAT simulation, complex I of the ETC was predicted to be active, however with a lower flux magnitude.

The model also predicted a subset of reactions implicated with amino acid metabolism to be active in this simulation that was not predicted to be active in the mouse BAT simulation. Instead of predicting flux through the r0595 reaction that is responsible for methionine and cysteine metabolism, the model predicted the consumption of uncoupled protons by 5-Aminolevulinate Synthase (ALASm) which metabolizes glycine into 5aop_m. The final reaction of this subsystem involved the Glycine-Cleavage Complex which converts glycine and lipoyl protein (lpro_m) into amino-methyl dihydrolipoyl protein. The remaining reactions that were predicted to metabolize uncoupled protons following UCP reaction optimization and specific to the human simulations were Malate dehydrogenase (MDMm), a reaction belonging to folate metabolism (MTHFCm) and finally a reaction involved in the urea cycle (G5SDym).

## 4 Discussion

We present mitoMammal, the first mitochondrial metabolic network reconstruction that serves for modelling mitochondrial metabolism for two species, mouse and human. MitoMammal contains two sets of GPR rules, one set of mouse genes, and another set of human genes, meaning the model can be constrained by integrating -omics data from these two organisms. Given the high similarity between mouse and human mitochondrial metabolism, we had the choice between two possible ways to model murine metabolism with -omics data-based constraints: either we would transform mouse gene identifiers to human and use the human MitoCore model for subsequent constraint-based modelling; or we could generate a mitochondrial metabolic model based on MitoCore that could be used for both species. We chose the latter, as it first makes the workflow for modelling mito-metabolism for the user straightforward; and second, it also allows the researcher to consider metabolic differences between the two organisms as each organism comes with its own set of GPR rules. We further added the DHODH reduction of CoQ following pyrimidine synthesis as this pathway was absent in MitoCore. As such, mitoMammal is the most comprehensive metabolic model of mammalian mitochondria to date.

To demonstrate the model’s ability to model mouse and human mitochondrial metabolism we first verified mitoMammal’s ability to capture realistic rates of ATP production. We then constrained mitoMammal by integrating proteomic data extracted from mouse cardiac tissue and optimized ATP production. Predicted fluxes included lactate production from pyruvate and the assimilation of pyruvate into the TCA cycle, the import of glycine into the mitochondria and the involvement of CV within OXPHOS to produce optimal ATP to support cardiomyocyte mitochondrial function. The model also predicted the reduction of CoQ by CI, yet fatty acid oxidation to support ATP synthesis was not predicted. These predictions are in agreement with data reported on immature cardiomyocytes, which express low levels of fatty acids and high levels of lactate in the blood that activates anaerobic glycolysis as the major source of ATP production (Karbassi *et al.* 2020).

We hypothesize that the reversal of CII in heart is an artefact due to missing values in the proteomics data we used. We found that several proteins that are part of the ETC were not detected in the dataset from (Hansen *et al.* 2024). We confirmed this further by using mouse bulk transcriptome data from the Tabula muris project (Schaum *et al.* 2020) from heart tissue of 18 months old mice, where flux through the respiratory chain was as expected and high, including a forward flux through CII (Supplementary Table S3). Given this experience, we hypothesize that the original MitoCore model was not used in combination with gene expression data, which left incorrect GPR rules undetected. The resulting predictions of the model also suggest that using constraints based on gene expression data is an excellent method to validate the correctness of GPR rules in genome-scale metabolic models, as it will reveal problems of the constructed model with respect to gene paralogs whose expression is restricted to specific tissues (the gene Ndufb11b, as an example, is only expressed in testis and, weakly, in the intestine).

In this simulation, glycine was predicted to be imported into mitochondria and converted to glutamate. Glycine has been shown to protect against doxirubicine induced heart toxicity in mice (Shosha *et al.* 2023) which validates this prediction, and highlights the important role of glycine metabolism in cardiomyocytes (Quintanilla-Villanueva *et al.* 2024) in sustaining steady-state metabolism. Glycine has been shown to increase the ATP content of mitochondria isolated from cardiac cells, which serves as another validation, however, in this simulation we chose to optimize ATP production, so understanding if glycine plays an essential role in mitochondrial metabolism to support optimal ATP yields requires further research, and suggests another application of how mitoMammal can further our knowledge in this respect.

Lactate is reported to fulfil important purposes that include providing an energy source for mitochondrial respiration, and being a major gluconeogenic precursor. As such, it is heavily involved in cellular signalling (Brooks 2020). Several basic and clinical studies have revealed the role that lactate plays in heart failure with the consensus that high blood lactate levels indicate poor prognosis for heart failure patients (Zymliński *et al.* 2018). Current research on this topic aims to target lactate production, regulate lactate transport, and modulate circulating lactate levels in an attempt to find novel strategies for the treatment of cardiovascular diseases. The in-depth knowledge gained by metabolic modelling with mitoMammal could also facilitate advances in this field.

To further demonstrate the usability of mitoMammal with alternative objective functions, and to highlight the ability of mitoMammal to model mouse and human metabolism, we integrated proteomic data extracted from the isolated mitochondria of mouse BAT (Fig. 4) and integrated transcriptomic data of human BAs (Fig. 5). For both simulations, we optimized the UCP reaction considering its central role in uncoupling electrons from the ETC and sustaining BAT metabolism (Hansen *et al.* 2024). This leads to the dissipation of the PMF across the inner mitochondrial membrane which is essential for BAT function. Despite modelling two species with different -omics datasets, modelling BA metabolism with either human transcriptome or mouse proteome data resulted in several similar flux predictions. One such prediction relates to the metabolism of hexadecanoic acid, also known as palmitic acid, which has been shown to increase BA differentiation, decrease inflammation and improve whole-body glucose tolerance in mice (Unno *et al.* 2018) and humans (Wade *et al.* 2021). These data validate the predictions of hexadecanoic acid metabolism in both simulations.

Elevated levels of proline have been measured in mammalian BA tissue (Okamatsu-Ogura *et al.* 2020) and elevated levels of proline dehydrogenase have also been associated with BA differentiation, and thermogenesis and are correlated with UCP1 activity (Li *et al.* 2023). In both these simulations, mitoMammal indeed predicted proline reduction of CoQ via proline dehydrogenase, which is in line with these published data. Furthermore, it has been proposed that CoQ reduction by proline dehydrogenase activates ROS production which then activates signalling pathways that facilitate hormone-independent lipid catabolism and support adipose tissue thermogenesis (Lettieri Barbato and Aquilano 2016, Chouchani *et al.* 2017).

Both simulations of BA metabolism predicted the reverse activity of CII. It has been experimentally demonstrated that CII can work in reverse in bacterial mitochondria (Maklashina *et al.* 1998) and mammalian mitochondria (Spinelli *et al.* 2021, Kumar *et al.* 2022). There is an increasing evidence that reversal of Complex II is relevant for brown adipocytes in mice. CII reversal has been experimentally verified in conditions where oxaloacetate correlates to a reverse CII activity in mice BAT (Som *et al.* 2023). The authors demonstrate that high UCP levels resulted with a reduced mitochondrial membrane potential, which then consequently lowered the NADH/NAD+ ratio, increased oxaloacetate accumulation and reversed CII. The authors proposed a physiology relevant role of CII reversal in regulating ROS production. Metabolic models predict steady-state metabolism, and thus without modification, cannot account for metabolite accumulation, but as observed in Fig. 4A we do predict an import of OAA within the mitochondria which again serves as model validation. Similarly, OAA is also predicted to be imported into the mitochondria following the integration of human BAT transcriptomic data (Fig. 5A).

We also observed differences in metabolic fluxes when comparing the predictions following human transcriptomic data and mouse proteomic data integration. Following human transcriptomic data integration, the model predicted the import of pyruvate into the mitochondria which was not predicted following mouse proteomic data integration. Instead, pyruvate was predicted to be converted to mercaptopyruvate (mercppyr). The simulation involving integrating human transcriptomic data also predicted the export of 5aopm which was not predicted when integrating mouse proteomic data. 5aopm is a precursor metabolite of the heme biosynthesis pathway and is required for adipocyte differentiation (Moreno‐Navarrete and Fernández‐Real 2024). Disrupted heme biosynthesis in human and mouse adipocytes has been shown to result in decreased adipogenesis, impaired glucose uptake, and reduced mitochondrial respiration (Handschin *et al.* 2005, Moreno‐Navarrete and Fernández‐Real 2024). These experimental discoveries of 5aopm therefore serve to further validate flux predictions following transcriptomic data integration and account for the misprediction associated with integrating proteomic data. Alanine was also predicted to be exported into the mitochondria for the human transcriptome simulation, yet the mouse proteomic simulation predicted the import of alanine. Alanine import (Rodríguez-Martín and Remesar 1991) and export (Frayn *et al.* 1991) into mammalian BAT tissue has been previously reported; however, the more comprehensive analysis reported by (Park *et al.* 2023) describes that alanine is an abundant circulating amino acid and functions as a nitrogen carrier where it is transported to the liver for nitrogen release. In their paper, the authors observed a net zero exchange flux and account for this to an equivalent uptake and release flux of alanine. As such, the model’s prediction of alanine import could be correct concerning mice metabolism (Fig. 4). Regarding human BAT metabolism, it is understood that accumulation of glutamate may increase the transamination of pyruvate to alanine (Legendre *et al.* 2020, Borkum 2023), which mitoMammal predicts, but much less is known of the fate of alanine and further research is necessary to validate the specific prediction of the directionality of alanine metabolism in human BATs.

One remaining difference between the predictions (shown in Figs 4B and 5B) is the activity of ATP synthase (CV) which was reported to operate in reverse following integration of RNA sequencing data and predicted to be inactive following integration of proteomic data from mice. MitoMammal represents the activity of CV as a Boolean representation of 14 genes that share an ‘AND’ relationship and so all 14 genes, or proteins need to be expressed to correctly produce all the individual subunits for a fully functional enzyme. For these GPRs, all 14 RNA sequencing transcripts were quantified, and because of the known reversibility of CV, our adapted E-Flux algorithm constrained the upper and lower bounds that correlated to the lowest transcript level of these 14 genes. As a consequence, mitoMammal in this simulation predicted the reverse activity of CV. For the proteomic simulation however, two of the 14 proteins were not identified (ENSMUSG00000000563; ENSMUSG00000064357) and an additional 4 proteins (ENSMUSG00000006057; ENSMUSG00000062683; ENSMUSG00000018770; ENSMUSG00000018770) were recorded as zero counts. As such, CV in this simulation was effectively constrained to zero and took no part in sustaining metabolic flows. ATP synthase (CV) is well known to operate in reverse during a wide range of different physiological environments to generate a mitochondrial membrane potential through ATP hydrolysis (Junge and Nelson 2015, Acin‐Perez *et al.* 2023) and the capacity of ATP hydrolysis has been observed in mitochondria isolated from BAT from mice (Acin‐Perez *et al.* 2023, Brunetta *et al.* 2024) and from humans (Harb *et al.* 2023). Reversal of ATP synthase in mice has recently been attributed to the activation of Inhibitory Factor 1 (IF1) (encoded by Atp5if1/ATP5IF1), which when activated, inhibits the reverse activity of ATP synthase. The work by (Brunetta *et al.* 2024) demonstrates that downregulation of IF1 is critical to support ATP hydrolysis, by allowing ATP synthase to operate in reverse, which then permits non-shivering thermogenesis in mouse BAT. As such, these findings serve to validate the predictions made following the integration of human transcriptomic data, and highlight limitations of proteomic data in terms of missing data, as discussed in (Vanderaa and Gatto 2021) and (Boys *et al.* 2023). We have indeed quantified this by observing the fact that integrating transcriptomic data resulted in constraining more reactions than proteomic data [489 reactions (BA, Human) versus 329 (BAT mouse) or 330 (Cardiac mouse)].

Regarding the other reactions of the ETC, human BAT transcriptomic data integration predicted the reduction of CoQ by G3PDH and proline, yet for the mouse simulation with proteomic data, proline reduced CoQ, and G3PDH was predicted to be inactive. G3PDH reduction of CoQ has been experimentally determined for BAT in both humans and mice (Banerjee *et al.* 2022, Oh *et al.* 2024). G3PDH is involved in the glycerol 3-phosphate shuttle, which, similarly to the MAS, shuttles reducing power in the form of NADH from the cytoplasm into the mitochondria. G3PDH then oxidizes the imported NADH into NAD+ and releases an electron which reduces CoQ. Both mouse and human BAT express high levels of G3PDH, and knockout of G3PDH in both species are associated with metabolic type 2 diabetes mellitus and obesity (Brown *et al.* 2002, Banerjee *et al.* 2022, Armani and Caprio 2023). Given this, we believe the prediction of an inactive G3PDH flux in mice associated with proteomic data integration to be a misprediction as the G3PDH protein abundance (ENSMUSG00000026827; 331) was identified in the dataset, and so the upper bound was constrained to a corresponding positive value and the lower bound was constrained to zero. We therefore attribute this error to the E-Flux methodology that only constrains the upper bound and neglects to constrain the lower bound. This, combined with linear programming to maximize an objective reaction meant that in the context of the mouse, the lower bound of zero associated to the G3PDH reaction was predicted to be used to optimize flux towards the UCP reaction, as such ignoring the reactions involvement to satisfy the objective. In the context of the human simulation that used transcriptomic data (Fig. 5A), the model predicted an activity of G3PDH to optimize the UCP reaction.

Some limitations have to be brought forward with this type of constraint-based modelling. For once, it is important to highlight the challenge associated with FBA of defining the correct biological objective reaction to optimize. While biomass as an approximation for bacterial growth is most likely justifiable in many cases, it is difficult to correctly assume a correct and foremost unique objective function for eukaryotic cells. There are promising developments in the field to circumnavigate this problem, including context specific multiobjective optimization (Liu and Westerhoff 2023), or avoidance of the objective completely by using flux-sampling methods (Herrmann *et al.* 2019, Galuzzi *et al.* 2023), whereby each method comes with its own set of challenges.

Another limitation of this study is that mitochondria within a cell are numerous, and here we are assuming that all mitochondria within one tissue conduct identical metabolism and operate independently from others, which may not be realistic. Mitochondrial activity is also influenced by crosstalk with organelles such as the golgi apparatus and endoplasmic reticulum. We chose here to specifically ignore this crosstalk in choosing the MitoCore model as a small and concise model that is capable of modelling mitochondrial metabolism. The contribution of other organelles is thus limited to observed imported and exported metabolites which, if experimentally known, can be used to constrain the model. One future research opportunity could be to establish small and precise models of other organelles, such as ER or peroxisomes, which then can be connected via import/export reactions.

Finally, we chose the E-Flux algorithm to integrate expression data with mitoMammal. As reviewed in (Blazier and Papin 2012), numerous methods of -omics data integration are available in addition to E-Flux. For example, gene Inactivity Moderated by Metabolism and Expression compares omic expression levels to a threshold to determine sets of active reactions in a metabolic model (Becker and Palsson 2008), while the Integrative Metabolic Analysis tool uses expression data to categorize reactions into high, moderate, or lowly active subsets (Shlomi *et al.* 2008). Both these methods incorporate expression data into metabolic models by reducing gene expression levels to discrete binary states. The E-Flux method however constrains the upper bound of a reaction to a continuous value that is relative to the expression level of the corresponding gene. Because of this, the E-Flux approach offers a more physiologically relevant method of data integration which is why we used this algorithm in this work. One related limitation, as reported with the original E-Flux method, is that the method only constrains the upper bounds of irreversible reactions, and for reversible reactions, sets the lower bound to a negative value of the upper bound, and assumes that expression of a gene is proportional to its activity. An algorithm that could constrain both the upper and lower reaction constraints would, therefore, turn this challenge into an opportunity by further reducing the solution space to yield more accurate predictions. A further limitation of this work relates to the concise nature of mitoMammal with its ability to integrate concise omics data specific to the mitochondria. For this, a good and complete dataset is required as incomplete data is not adequate to fully constrain the model. This includes for instance accurately inferred gene expression data of mitochondria-encoded genes.

We have demonstrated that mitoMammal can be used with different objective functions which is a crucial step in constraint-based metabolic modelling (Dikicioglu *et al.* 2015, Schnitzer *et al.* 2022). In our simulations of heart metabolism, as a consequence of optimizing maximum ATP production, metabolic flux was predicted to avoid the UCP reaction. This prediction has also been experimentally validated in the work of (Hansen *et al.* 2024) who show that the UCP1 protein is inactive for cardiac tissue, yet active in BAT cells which highlights the metabolic flexibility of mitochondria in supporting tissue-specific function.

## Supplementary Material

vbae172_Supplementary_Data

## Data Availability

The data underlying this article are available in gitlab at https://gitlab.com/habermann_lab/mitomammal, and can be accessed without restriction.
