# Supplementary material for: MitoMAMMAL: a genome scale model of mammalian mitochondria predicts cardiac and BAT metabolism
Source: Bioinform Adv. 2024 Nov 5;5(1):vbae172. doi: 10.1093/bioadv/vbae172 (PMC11696703; doi:10.1093/bioadv/vbae172)
Supplement: vbae172_Supplementary_Data [file vbae172_supplementary_data.zip › Chapman_etal_SupplementaryMaterials.pdf]

Supplementary materials

Supplementary Figure S1:

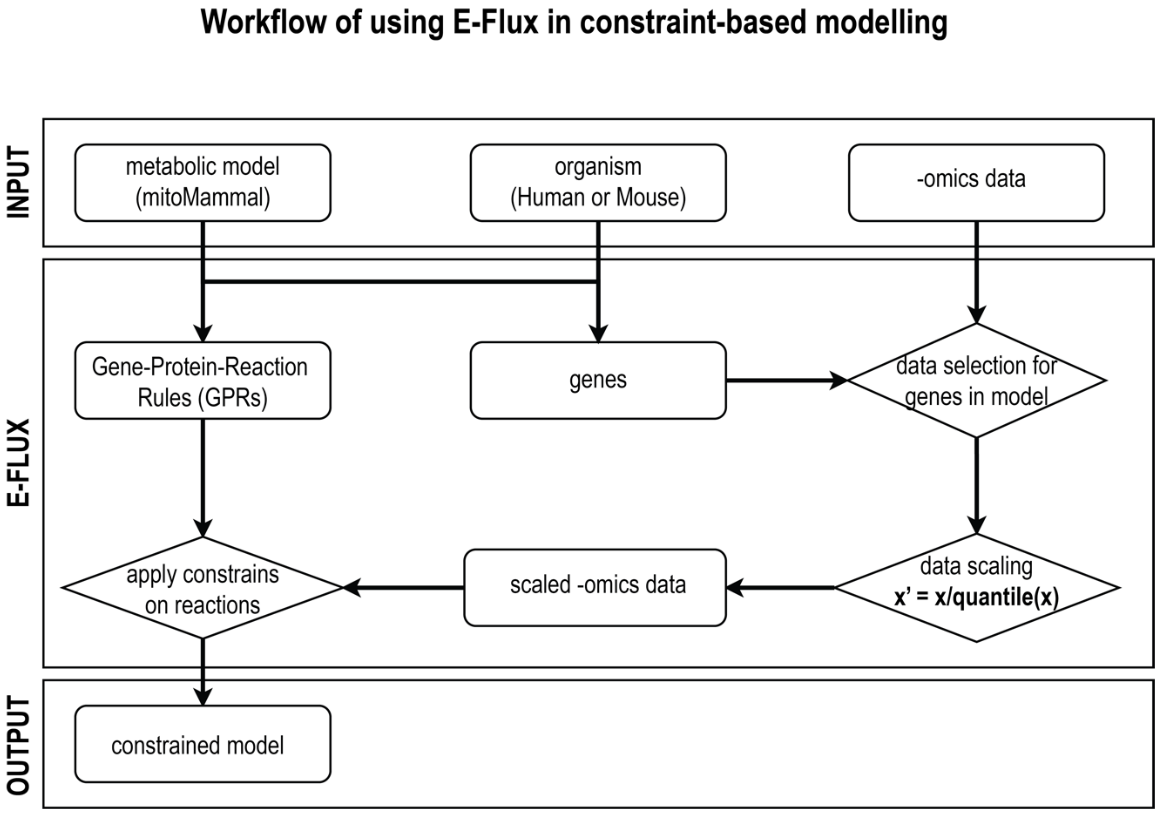

Supplementary Figure S1: Workflow of using E-Flux in constraint-based modelling. The user needs to provide a metabolic model, the choice of an organism (human or mouse), as well as -omics data (proteomic or transcriptomic). First, genes present in the model are selected from the -omics data and scaled by division by the quantile. The resulting scaled values are used to constrain the reactions in the metabolic model. In case of AND relationships in GPRs, the lowest expressed gene is used as upper constraint. In case of OR relationships in GPRs, the sum of all involved genes from the GPR is used as upper constraint.

Supplementary Figure S2:

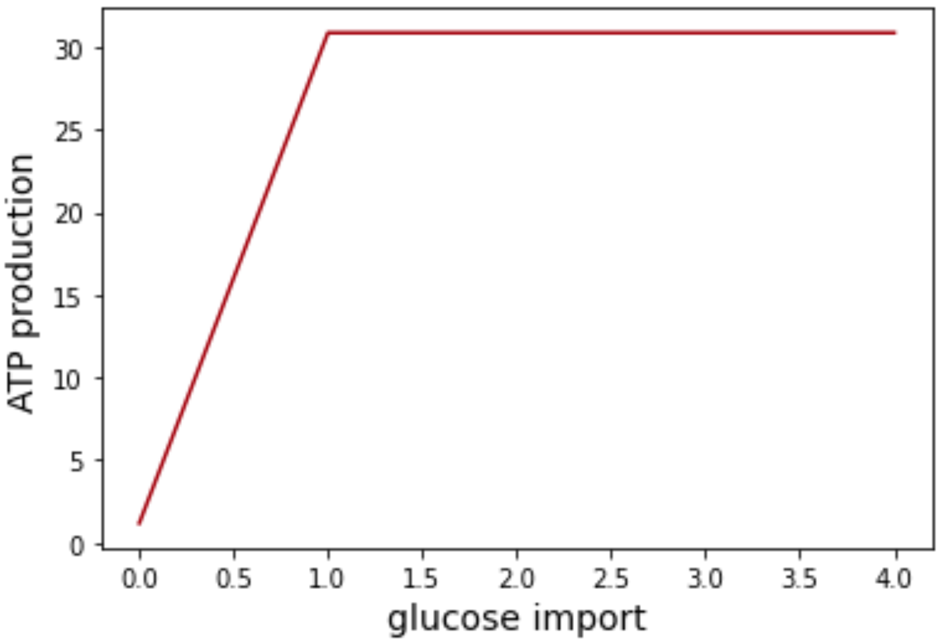

Supplementary Figure S2. 1 molecule of glucose import produces 31 molecules of ATP. By convention import fluxes are negative but to improve the comprehension we present the glucose import as positive values.

Supplementary tables:

Supplementary Table S1. A complete list of reactions, metabolites and associated fluxes presented in this manuscript are presented in Supplementary Tables S1a, S1b and S1c respectively (file Chapman_etal_SupplTableS1.xlsx).

Supplementary Table S2: reactions that consume hydrogen following the optimisation of the UCP1 reaction with integrated mouse proteomic data and human transcriptomic data.

Supplementary Table S3: Comparison of predictions of mouse heart, using proteomics (Hansen, et al., 2024) and transcriptomics data at 18 weeks from the Tabula muris project (Schaum, et al., Nature 2020).
